# Preservation of Mitochondrial Coupling and Renal Function by Controlled Oxygenated Rewarming of Porcine Kidney Grafts

**DOI:** 10.3390/biom11121880

**Published:** 2021-12-14

**Authors:** Hristo Zlatev, Charlotte von Horn, Thomas Minor

**Affiliations:** Surgical Research Department, Clinic for General, Visceral and Transplantation Surgery, University Hospital Essen, Hufelandstr. 55, D-45147 Essen, Germany; chirfor@uk-essen.de (H.Z.); charlotte.von-horn@uk-essen.de (C.v.H.)

**Keywords:** controlled oxygenated rewarming, mitochondrial uncoupling, rewarming injury, temperature paradox

## Abstract

Background: Warm reperfusion after previous cold storage has been shown to have a negative impact on mitochondrial function of organ grafts. Here, we wanted to investigate whether a more controlled warming up of the cold graft by ex vivo machine perfusion with gradually elevated temperature from cold to normothermia (including comparison of two warming up protocols) prior to implantation would be effective in preventing mitochondrial dysfunction upon reperfusion. Methods: All experiments were conducted on porcine kidneys retrieved 15 min after cardiac arrest. After 18 h of cold storage in HTK solution (CS, n = 6), kidneys (n = 6) were subjected to 2 h of reconditioning machine perfusion starting with a hypothermic period followed by a gradual increase in perfusion temperature up to 35 °C (controlled oxygenated rewarming—COR). For a second group (n = 6), the slow warming up was begun instantly after connecting the graft onto the machine (iCOR). Functional recovery of all grafts was then observed upon normothermic reperfusion in vitro. At the conclusion of the experiments, tissue specimens were taken for immediate isolation and analysis of renal mitochondria. Results: COR resulted in a significantly and more than 3-fold increased glomerular filtration rate upon reperfusion, along with a significant higher tubular sodium reabsorption and lesser loss of glucose in comparison to the controls. Enzyme release (AST) was also massively reduced during the reperfusion period. Specific analysis at the mitochondrial level revealed significantly better coupling efficiency and spare respiratory capacity in the COR group compared to the cold storage group. Interestingly, additional experiments revealed that the omission of a hypothermic perfusion period did not deteriorate any of the results after COR, provided that the instant temperature increase from 10 to 35 °C was effectuated in the same controlled manner. Conclusion: Controlled rewarming after extended cold preservation effectively improves mitochondrial recovery upon reperfusion and early functional outcome of kidney grafts.

## 1. Introduction

Organ damage resulting from preservation or reperfusion still represents a major issue in transplantation medicine. Extended preservation times or graft donation after cardiocirculatory standstill in the donor are often conflicted with a reduced recovery after transplantation affecting early graft function, as well as long-term survival.

Although the underlying mechanistic pathways of preservation injury are not yet fully understood, altered mitochondrial integrity upon reperfusion has been deciphered as a major culprit for graft dysfunction after transplantation [1,2]. During cold ischemic preservation, opening of the mitochondrial transition pore (MTP) could be observed along with mitochondrial swelling [3,4]. However, mitochondrial respiratory capacity has been shown to remain stable for up to 24 h of cold storage and only deteriorates after unusual prolongation of cold ischemia or upon subsequent warm reperfusion [5].

Likewise, cytosolic release of cytochrome c and sequential induction of apoptosis are only observed after subsequent rewarming [4,6,7]. In line with these observations, main parts of organ preservation injury substantiate not during cold storage but upon and in consequence of abrupt rewarming [8,9]. Hence, significant alleviation of functional impairment of kidney grafts after transplantation could be achieved by modifying the rise in temperature during reoxygenation using a controlled oxygenated rewarming (COR) protocol subsequent to cold storage by thermoregulated machine perfusion [10,11]. The use of an incremental temperature rise instead of an abrupt return to normothermia improved post-ischemic recovery of discarded human donor livers [12] and resulted in superior results after experimental [10] and clinical [13] kidney transplantation. COR was also found to be operative in significantly reducing the initiation of the mitochondrial apoptotic pathway upon reperfusion along, with a better preservation of mitochondrial content of NAD+ [14]. Likewise, COR improved global recovery of tissue energetics [9] and largely preserved oxygen utilization efficiency at the whole organ level that was otherwise found significantly disturbed [11].

Controlled oxygenated rewarming during machine perfusion classically comprises three phases. A first period of oxygenated cold perfusion is undertaken to fuel residual aerobic metabolism to ameliorate subcellular homeostasis while avoiding major temperature shifts [15,16]. Then follows a thermal transition phase of slow and adapted increase in the perfusion temperature [9] and a final steady state period of perfusion at the final temperature for a variable time span [7]. 

Although the positive effects of a brief hypothermic perfusion period alone have been thoroughly established in that the cellular aerobic energetic homeostasis could be improved in the cold and that the tissue is better prepared for the following abrupt warm reperfusion [15,16,17], the relevance of an extended cold perfusion phase versus an immediate start of the gentle warming up protocol in the setting of COR has not yet been addressed. 

Thus, the aim of the present study was to scrutinize the relative impacts of a hypothermic equilibration phase in the setting of a controlled rewarming protocol with a special focus on mitochondrial function and coupling status.

## 2. Materials and Methods

All experiments were performed in accordance with the federal law regarding the protection of animals. The principles of laboratory animal care (NIH publication no. 85-23, revised 1985) were followed. 

**Figure 1:**
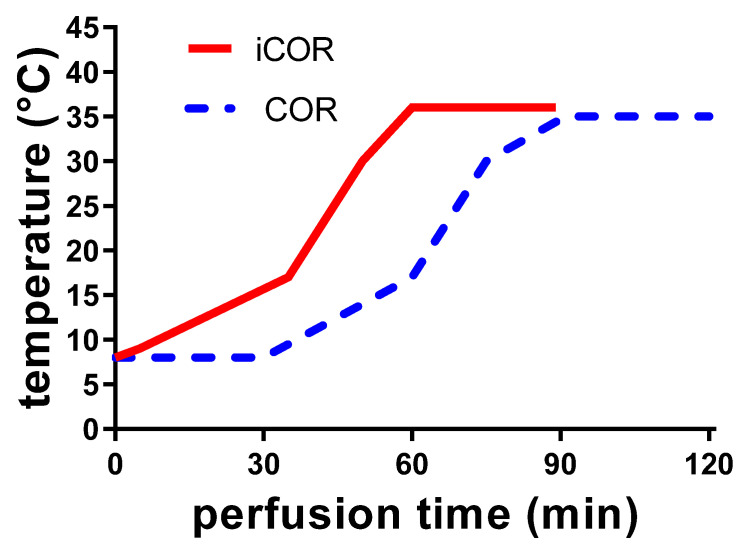
Representation of the thermal kinetic during controlled oxygenated rewarming by machine perfusion of porcine kidney grafts. Gentle rewarming of kidneys is either performed after an initial 30 min hypothermic equilibration period (COR, hatched line) or by starting the rewarming protocol immediately after connection to the machine (iCOR, continuous line).

Kidneys were removed from dead German Landrace pigs weighing between 25 and 30 kg. Then, 15 min after cardio-circulatory standstill, the renal artery was cannulated and the kidneys were flushed by 100 cm gravity with 100 mL of HTK solution (Köhler Chemie, Bensheim, Germany) on the back-table at 4 °C. No heparin was given at any time. After 18 h of static cold preservation in HTK solution, the grafts were randomly assigned to one of the following groups (n = 6, resp.):

1. In the control group, kidneys were exposed to 18 h of static cold storage at 4 °C without additional treatment (CS);
2. In the second group, controlled oxygenated rewarming (COR) was performed after 18 h of CS by end-ischemic machine perfusion, as described earlier [11,18]. Perfusion with Aqix-RS-I (Life Science Group, Bedford, UK) was started at a temperature of 8 °C with consecutive rewarming of the perfusate from 8 °C to 35 °C during the first 90 min. The rise in temperature was accompanied by an adapted increase of the perfusion pressure from 30 to 75 mmHg. The last 30 min of perfusion was kept constant at 35 °C (cf. Figure 1);
3. A third group was investigated, whereby the slow warming up was begun instantly after connecting the graft onto the machine, i.e., omitting the hypothermic equilibration phase (cf. Figure 1).

Perfusion with Aqix-RS-I (Life Science Group, Bedford, UK) was started at a temperature of 8 °C but instant controlled oxygenated rewarming (iCOR) was started increasing temperature up to 35 °C during the first 60 min in a hyperbolic pattern comparable to the middle period in group 2. The rise in temperature was accompanied by an adapted increase of the perfusion pressure from 30 to 75 mmHg. The last 30 min of perfusion was kept constant at 35 °C.

### 2.1. Reperfusion Model

Prior to reperfusion all grafts were flushed with 100 mL of cold saline solution and exposed to no flow conditions at room temperature for 20 min in order to imitate the time of surgical engraftment.

The functional recovery of the grafts was tested using an established in vitro model, as previously described [19], which was modified regarding the replacement of fluid loss by urine production during ongoing reperfusion [20].

In brief, kidneys were put into a moist chamber and perfused at 37 °C with 1000 mL Krebs–Henseleit buffer to which were added 2.2% bovine serum albumin (PAN-Biotech, Aidenbach, Germany) and 20 mL of concentrated amino acid solution (RPMI 1640 Amino Acids Solution, 50×, PAN-Biotech, Aidenbach, Germany). 

Perfusate was oxygenated with a mixture of 95% oxygen and 5% carbon dioxide by a hollow fiber oxygenator (Hilite LT 1000, Medos, Stolberg, Germany) and supplemented with 0.05 g/L of creatinine to allow for calculation of renal clearances. Cannulation of the ureter was performed with PE tubing for urine collection throughout the reperfusion period. 

Urine produced during reperfusion was collected and reinfused after filtration (13 µm) at 100 mL intervals to the reservoir in order to prevent alterations in the composition of the perfusate over time, which would be encountered when replacing urine loss by adding balanced salt solution [21]. The volume of urine production was measured for each individual 30 min interval and representative aliquots from each fraction were pooled for the respective intervals for later analysis of metabolites. 

Kidney perfusion pressure was set at 90 mmHg and automatically maintained by a servo-controlled roller pump connected to a pressure sensor placed in the inflow line immediately prior to the renal artery.

At the end of the perfusion, kidneys were removed from the perfusion chamber and samples were taken for biochemical analysis and for isolation of mitochondria.

### 2.2. Mitochondria Isolation and Analysis

The mitochondria were isolated as described in [22]. Mitochondria isolation buffer (MIB) was prepared with final concentrations of 70 mM sucrose, 210 mM mannitol, 53 mM HEPES, and 1 mM EGTA, (pH adjusted to 7.4). Mitochondrial assay buffer (MAS) was composed of 70 mM sucrose, 220 mM mannitol, 10 mM KH_2_PO_4_, 2 mM MgCl_2_, 2 mM HEPES, 1 mM EGTA, and 0.2% *w*/*v* fatty-acid-free BSA (pH 7.4).

The mitochondrial oxygen consumption rate (OCR) and extracellular acidification rate (ECAR) were assessed using a Seahorse XFe24 Analyzer (Agient Technologies, Santa Clara, CA, US) and Seahorse XFe24 FluxPack (Agilent Technologies, Santa Clara, CA, US) as described by Rogers et al. (11). 

Mitochondria were incubated with substrate (2 mM rotenone and 0.5 M succinate), following by sequential addition of 1M adenosine 5′-diphosphate potassium salt (ADP), 5 mg/mL oligomycin (ATP synthase inhibitor), 10 mM carbonyl cyanide-4-(trifluoromethoxy) phenylhydrazone (FCCP, mitochondrial uncoupler), and 40 mM antimycin A (complex 3 inhibitor). All substances were purchased from Sigma-Aldrich, Germany. The results were analyzed with Wave Seahorse Software (Agilent Technologies, Santa Clara, CA, USA).

### 2.3. Analytical Procedures

Analytical routines were performed as described previously [20]. The activities of aspartate aminotransferase (AST) and concentrations of creatinine were determined in a routine fashion by reflectance photometry on a Reflotron Plus point of care unit (Roche Diagnostics, Mannheim, Germany).

Clearances were calculated for the respective intervals as urinary creatinine x urine flow/perfusate creatinine.

The albumin concentration in urine was measured in a routine fashion at the Laboratory Center of the University Hospital and the amount of protein normalized against the corresponding concentrations of creatinine as the urinary albumin-to-creatinine ratio (mg/mg).

Oxygen partial pressure and perfusate concentrations of sodium were measured in a pH-blood gas analyzer (ABL 815flex acid-base laboratory, Radiometer, Copenhagen). 

Oxygen consumption (VO_2_) was calculated from the differences between arterial and venous sites and expressed as mL min^−1^ g^−1^ according to the trans-renal flow and kidney mass.

The efficiency of renal O_2_ utilization was approximated by the ratio of total kidney transport of Na (TNa), accounting for the vast majority of energy consuming processes in the kidney [23], and VO_2_, with TNa being equal to filtered Na minus excreted Na:

TNa = (GFR × Perfusate Na) − (urinary Na × urine flow)

Fractional excretion of sodium (FE Na^+^) was calculated according to:

FE Na^+^= Na^+^_(urine)_ × Creatinine_(perfusate)_/Na^+^_(perfusate)_ × Creatinine _(urine)_ × 100

Measurement of neutrophil-gelatinase-associated lipocalin (NGAL) was performed with a commercialized ELISA kit (USCN life science, Wuhan, China) according to the instructions of the manufacturer on a fluorescence microplate reader (Tecan, Grailsheim, Germany).

### 2.4. Histology

At the conclusion of the experiment, tissue samples were collected for later histological examination. Specimens were cut into 3 mm blocks, fixed by immersion in 4% buffered formalin, and embedded in paraffin. Tissue slides were prepared on a SM 2000R microtome (Leica Instruments, Nußloch, Germany). Light microscopy (20× magnification) of periodic acid Schiff-stained sections were used to demonstrate changes in morphology.

### 2.5. Statistical Analysis

Kidneys were randomized with n = 6 kidneys per group. Results are expressed as means ± standard deviation. Stochastic significance of differences was assessed using one way analysis of variance and Dunn’s multiple comparisons test, if not otherwise indicated.

Data were analyzed with GraphPad Prism version 8.0.0 (GraphPad Software, San Diego, CA, USA, www.graphpad.com). Significance was defined as *p* < 0.05.

## 3. Results

**Figure 2:**
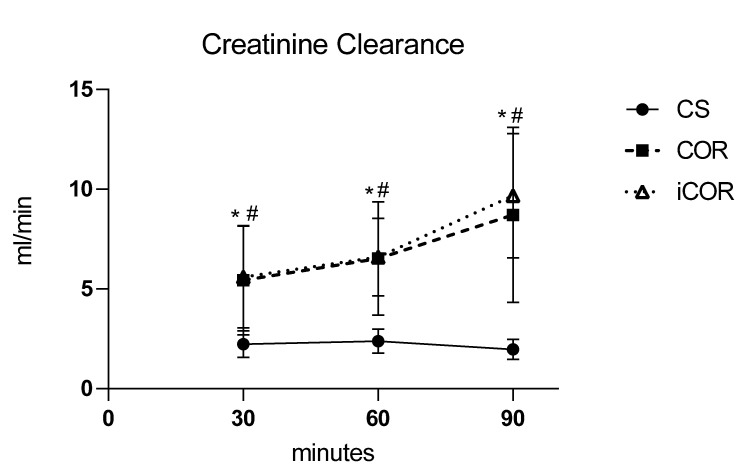
Clearance of creatinine during reperfusion in vitro after 20 h cold storage (CS) or after 2 h of subsequent controlled oxygenated rewarming with (COR) or without (iCOR) an initial hypothermic perfusion period (*: *p* < 0.05 COR vs. CS; #: *p* < 0.05 iCOR vs. CS).

The glomerular filtration function was evaluated by measurement of the clearance of creatinine during warm reperfusion (cf. Figure 2). Controlled oxygenated rewarming (COR) was followed by a significant enhancement of glomerular filtration rate when compared to cold-stored controls. Of note, omitting the hypothermic equilibration phase in the instant rewarming (iCOR) group did not result in any adverse effect in comparison to the COR group.

Likewise, urinary leakage of albumin (expressed as the albumin/creatinine ratio) was markedly increased to 0.40 ± 0.14 in cold-stored kidneys but significantly (*p* < 0.05) reduced after COR (0.13 ± 0.05), as well as after iCOR treatment (0.12 ± 0.03) prior to reperfusion.

Tubular cell integrity was also found to be protected by both of the rewarming protocols. The concentration of neutrophil-gelatinase-associated lipocalin (NGAL) in the circulating perfusate was used as an indicator of tubular cell stress. It amounted to 6.8 ± 0.6 ng/mL in the cold storage group but was significantly (*p* < 0.05) reduced to 3.4 ± 0.6 and 3.9 ± 1.1 ng/mL in the COR and iCOR groups, respectively. 

Differences were even more pronounced regarding the enzyme leakage of AST into the perfusate, which amounted to 659 ± 328 U/L, 140 ± 73 U/L * and 182 ± 249 U/L * after CS, COR, and iCOR, resp. (*p* < 0.05).

**Figure 3:**
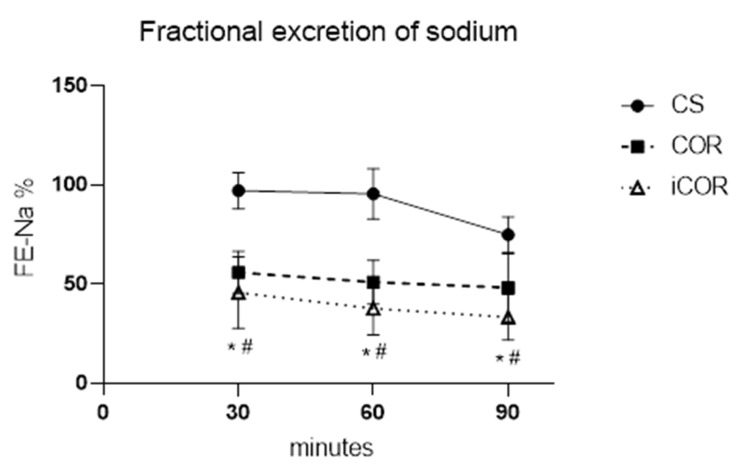
Course of fractional excretion of sodium (FENa) during isolated reperfusion in vitro after 20 h cold storage (CS) or after 2 h of subsequent controlled oxygenated rewarming with (COR) or without (iCOR) an initial hypothermic perfusion period (*: *p* < 0.05 COR vs. CS; #: *p* < 0.05 iCOR vs. CS).

Tubular cell function was followed by measuring the fractional excretion of sodium from the renal ultrafiltrate (cf. Figure 3). Large amounts of the filtrated sodium could not be re-absorbed and were, thus, excreted with the urine in the cold storage group, although the excretion rate slightly improved during ongoing reperfusion. In contrast, significantly better values were observed after controlled oxygenated rewarming, and this benefit was independent from the inclusion of a hypothermic starting period during the rewarming protocol.

**Figure 4:**
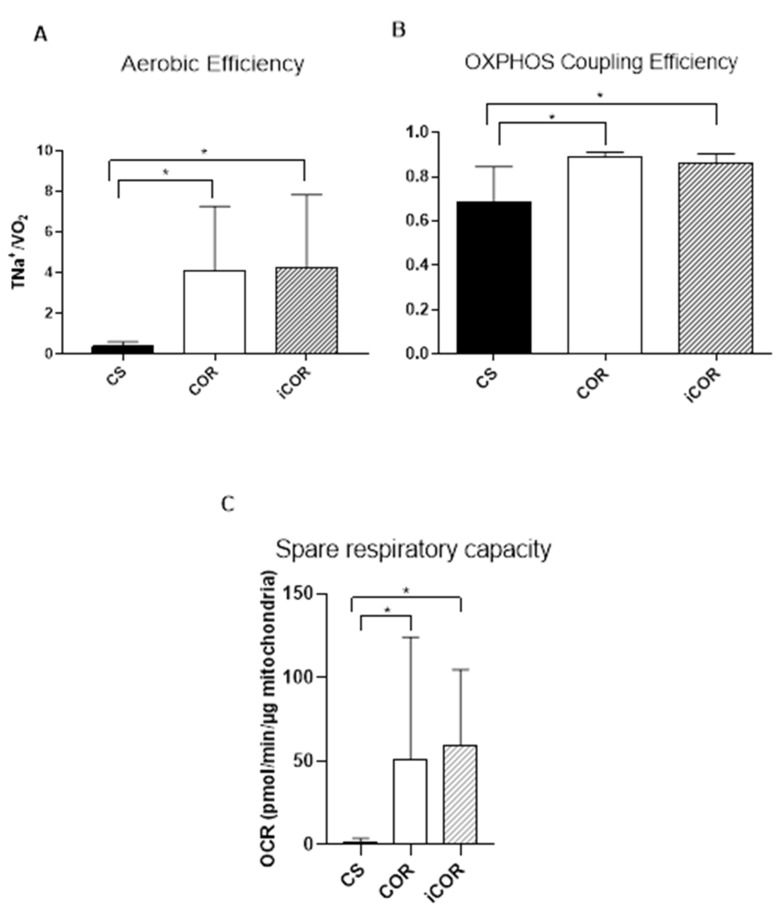
Parameters of mitochondrial function upon isolated reperfusion in vitro after 20 h cold storage (CS) or after 2 h of subsequent controlled oxygenated rewarming with (COR) or without (iCOR) an initial hypothermic perfusion period: (**A**) renal aerobic efficiency reflected by the ratio of total tubular sodium transport (TNa) and oxygen consumption (VO2); (**B**) OXPHOS coupling efficiency and (**C**) spare respiratory capacity of isolated mitochondria at the end of the 90 min reperfusion period (*: *p* < 0.05 vs. CS).

The efficiency of oxygen utilization by the renal tissue is approximated by the ratio between total sodium transport (TNa), accounting for the vast majority of renal energy consumption, and corresponding oxygen consumption (VO_2_). Cold-stored kidneys showed a markedly reduced TNa/VO_2_ ratio upon reperfusion, indicating a massively disturbed aerobic efficiency (Figure 4A). However, this impairment of oxygen utilization efficiency was significantly ameliorated by using one of the controlled rewarming protocols prior to reperfusion. Again, the protective effects of both rewarming protocols were virtually identical.

Specific mitochondrial function upon reperfusion was evaluated by respiratory assays with freshly isolated mitochondria from renal tissue at the end of reperfusion. The coupling assay reflects the oxidative phosphorylation efficiency of mitochondria. 

In line with the data on oxygen utilization efficiency at the whole organ level, mitochondrial coupling efficiency was notably compromised in the CS group (Figure 4B), whereas controlled rewarming using either the COR or the iCOR protocol resulted in a significantly better preserved oxidative electro-chemical coupling rate at the mitochondrial level.

Spare respiratory capacity (SRC) characterizes mitochondrial reserve to meet an increasing energy demand in response to stress conditions. Mitochondria of only cold-stored kidneys showed nearly no functional reserve upon warm reperfusion, whereas mitochondria of COR or iCOR treated kidneys yielded considerably higher SRC values. 

Significant differences between the two rewarming protocols regarding mitochondrial function could not be observed in this setting (Figure 4C).

**Figure 5:**
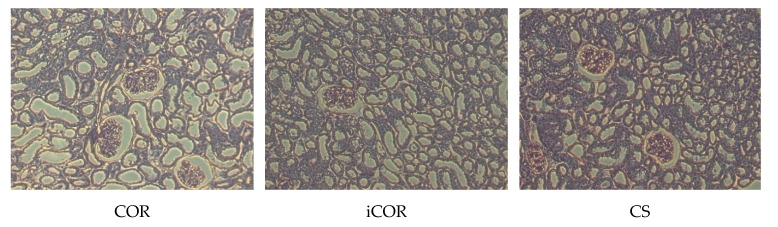
Representative sections of kidney tissue after reperfusion subsequent to preservation by either cold storage with controlled oxygenated rewarming (COR), instant controlled oxygenated rewarming (iCOR), or by cold storage without controlled rewarming (CS). PAS staining, 20× original magnification. Note the reduced tubular cell vacuolization and absence of necrosis in the groups COR and iCOR.

Light microscopy performed on tissue samples obtained after conclusion of the experiment did not disclose any glomerular damage, with only mild alterations in either group. However, some alterations of normal structural appearance were observed, mainly comprising tubular cell vacuolization, which was notably less prominent after COR or iCOR than in the cold storage group. Sporadic signs of necrosis were also observed only after cold storage (Figure 5).

## 4. Discussion

Gentle elevation of tissue temperature after extended periods of hypothermia during cold preservation has been shown to favor a significantly more thorough restitution of mitochondrial function. Concomitantly, the whole-graft outcome during early reperfusion could be notably improved. 

The latter observation is in line with previous reports, indicating controlled oxygenated rewarming to mitigate rewarming injury upon reperfusion in vitro [11], in vivo [10], and in clinical kidney transplantation [13].

Our present study, however, went further. We disclosed a notable reduction in the ratio of total sodium absorption and oxygen consumption (TNa/VO2) after cold storage and reperfusion that was significantly ameliorated by way of gentle warming up of the grafts prior to reperfusion. Thus, oxygen expenditure is only incompletely met by useful endergonic metabolism. At first sight, this seems to be attributable to an increasing uncoupling phenomenon at the mitochondrial level triggered upon warm reperfusion after cold preservation and significantly attenuated upon only slow elevation of temperature. 

Another possible explanation for the reduced TNa/VO2 ratio might relate to oxygen-consuming cellular repair processes or resurrection of brush border microvilli [24]. Here, we provide actual direct evidence for mitochondrial uncoupling during the early reperfusion period after cold preservation. In addition, spare respiratory capacity, i.e., the amount of extra ATP that can be produced by oxidative phosphorylation in case of a sudden increase in energy demand, is largely depressed after cold storage. Hence, mitochondrial dysfunction rather than deviant oxygen utilization is responsible for the reduced TNa/VO2 ratio. 

Interestingly, controlled oxygenated rewarming is operative in preserving mitochondrial performance upon reperfusion after hypothermic preservation, and this effect was not dependent on a longer hypothermic perfusion phase at the start of the rewarming protocol. This is in ostensible contrast to previous work from others, as well as from us, which consistently shows that a short oxygenated hypothermic perfusion prior to transplantation effectively improves the graft’s resilience to reperfusion injury [15,16,25]. In contrast, hypothermic machine perfusion partly restores energetic and metabolic tissue homeostasis and helps to mitigate ischemia or reperfusion injury. However, even satisfactory aerobic conditions in the cold cannot prevent the adverse effects of an abrupt thermal transition to normothermia and a dramatic impairment of the mitochondrial respiratory control ratio [8]. 

Previous comparative studies have shown that although brief hypothermic machine perfusion improved functional outcome of cold-stored kidney [14] and liver grafts [9], notably better protection was provided by additional controlled oxygenated rewarming. Apparently, even energized tissue remains susceptible to the temperature paradox phenomenon that incurs along with the abrupt rewarming. On the other hand, slowing down of the rewarming process alone appears to be sufficient to allow for a graduated resumption of subcellular, especially mitochondrial homeostasis adapted to physiological demands [7].

In clinical practice, the process of connecting the graft to the machine and starting the ex vivo perfusion is more easily done in the cold, when hypothermia of the perfusate and graft allows for meticulous cannulation of the vessels without danger of normothermic tissue ischemia in the machine. Sometimes, however, limitation of the total perfusion time on the machine may be desirable, e.g., if the recipient is readily prepared or the total preservation time should be kept at a minimum. In these cases, it will, thus, be a suitable measure to start the rewarming protocol in the cold but to initiate the graduated rise in temperature immediately after organ connection has been effectuated and spare up to 30 min of perfusion in the cold.

The well-known limitations of all in vitro models also apply to our study. In contrast to cell culture experiments, which do not allow for functional evaluation of the whole organ, our model strictly mimics the clinical ex vivo graft perfusion and provides functional data on early reperfusion outcome at the whole organ level. Although no long term evaluation of graft function has been possible in the present study, nor could interferences of whole blood with the post-ischemic tissue be accounted for, close similarities of functional data after COR could be found between the present study and earlier investigations in vivo [10]. Apart from this, the primary goal of the present study, i.e., to decipher the mechanistic role of early mitochondrial dysfunction, triggered upon abrupt warm reperfusion after cold storage and its prevention by controlled oxygenated rewarming could be sensibly addressed by the used model.

In conclusion, our data provide evidence for mitochondrial uncoupling upon abrupt warm reperfusion after extended cold preservation of renal rafts, which can effectively be alleviated by controlling the rewarming process.

## Data Availability

Data are available from the authors upon reasonable request.

## References

[1] Schlegel A., Muller X., Mueller M., Stepanova A., Kron P., de Rougemont O., Muiesan P., Clavien P.A., Galkin A., Meierhofer D. (2020). Hypothermic oxygenated perfusion protects from mitochondrial injury before liver transplantation. EBioMedicine.

[2] Saeb-Parsy K., Martin J.L., Summers D.M., Watson C.J., Krieg T., Murphy M.P. (2021). Mitochondria as Therapeutic Targets in Transplantation. Trends Mol. Med..

[3] Saris N.E., Eriksson K.O. (1995). Mitochondrial dysfunction in ischaemia-reperfusion. Acta Anaesthesiol. Scand..

[4] Salahudeen A.K. (2004). Cold ischemic injury of transplanted kidneys: New insights from experimental studies. Am. J. Physiol. Ren. Physiol..

[5] Sammut I.A., Burton K., Balogun E., Sarathchandra P., Brooks K.J., Bates T.E., Green C.J. (2000). Time-Dependent Impairment of Mitochondrial Function After Storage and Transplantation of Rabbit Kidneys1. Transplantation.

[6] Duval M., Plin C., Elimadi A., Vallerand D., Tillement J.P., Morin D., Haddad P.S. (2006). Implication of mitochondrial dysfunction and cell death in cold preservation--warm reperfusion-induced hepatocyte injury. Can. J. Physiol. Pharmacol..

[7] Minor T., von Horn C. (2019). Rewarming Injury after Cold Preservation. Int. J. Mol. Sci..

[8] Leducq N., Delmas-Beauvieux M.C., Bourdel-Marchasson I., Dufour S., Gallis J.L., Canioni P., Diolez P. (1998). Mitochondrial permeability transition during hypothermic to normothermic reperfusion in rat liver demonstrated by the protective effect of cyclosporin A. Biochem. J..

[9] Minor T., Efferz P., Fox M., Wohlschlaeger J., Lüer B. (2013). Controlled oxygenated rewarming of cold stored liver grafts by thermally graduated machine perfusion prior to reperfusion. Am. J. Transplant..

[10] Von Horn C., Zlatev H., Kaths M., Paul A., Minor T. (2021). Controlled Oxygenated Rewarming Compensates for Cold Storage-induced Dysfunction in Kidney Grafts. Transplantation.

[11] Von Horn C., Minor T. (2018). Improved approach for normothermic machine perfusion of cold stored kidney grafts. Am. J. Transl. Res..

[12] Boteon Y.L., Laing R.W., Schlegel A., Wallace L., Smith A., Attard J., Bhogal R.H., Neil D.A., Hübscher S., Perera M.T.P. (2018). Combined Hypothermic and Normothermic Machine Perfusion Improves Functional Recovery of Extended Criteria Donor Livers. Liver Transplant..

[13] Zlatev H., von Horn C., Kaths M., Paul A., Minor T. (2021). Clinical Use of Controlled Oxygenated Rewarming of Kidney Grafts Prior to Transplantation by Ex Vivo Machine Perfusion: A Pilot Study. Eur. J. Clin. Investig..

[14] Schopp I., Reissberg E., Lüer B., Efferz P., Minor T. (2015). Controlled Rewarming after Hypothermia: Adding a New Principle to Renal Preservation. Clin. Transl. Sci..

[15] Stegemann J., Minor T. (2009). Energy charge restoration, mitochondrial protection and reversal of preservation induced liver injury by hypothermic oxygenation prior to reperfusion. Cryobiology.

[16] Kron P., Schlegel A., de Rougemont O., Oberkofler C.E., Clavien P.A., Dutkowski P. (2016). Short, Cool, and Well Oxygenated—HOPE for Kidney Transplantation in a Rodent Model. Ann. Surg..

[17] Minor T., Paul A. (2013). Hypothermic reconditioning in organ transplantation. Curr. Opin. Organ. Transplant..

[18] Minor T., von Horn C., Gallinat A., Kaths M., Kribben A., Treckmann J., Paul A. (2020). First-in-man controlled rewarming and normothermic perfusion with cell-free solution of a kidney prior to transplantation. Am. J. Transplant..

[19] Von Horn C., Minor T. (2018). Isolated kidney perfusion: The influence of pulsatile flow. Scand. J. Clin. Lab. Investig..

[20] Minor T., von Horn C., Paul A. (2019). Role of erythrocytes in short-term rewarming kidney perfusion after cold storage. Artif. Organs.

[21] Nizet A. (1975). The isolated perfused kidney: Possibilities, limitations and results. Kidney Int..

[22] Rogers G.W., Brand M.D., Petrosyan S., Ashok D., Elorza A.A., Ferrick D.A., Murphy A.N. (2011). High Throughput Microplate Respiratory Measurements Using Minimal Quantities of Isolated Mitochondria. PLoS ONE.

[23] Pei L., Solis G., Nguyen M.T., Kamat N., Magenheimer L., Zhuo M., Li J., Curry J., McDonough A.A., Fields T.A. (2016). Paracellular epithelial sodium transport maximizes energy efficiency in the kidney. J. Clin. Investig..

[24] Herminghuysen D., Welbourne C.J., Welbourne T.C. (1985). Renal sodium reabsorption, oxygen consumption, and gamma-glutamyltransferase excretion in the postischemic rat kidney. Am. J. Physiol..

[25] Gallinat A., Paul A., Efferz P., Lüer B., Kaiser G., Wohlschlaeger J., Treckmann J., Minor T. (2012). Hypothermic Reconditioning of Porcine Kidney Grafts by Short-Term Preimplantation Machine Perfusion. Transplantation.

